# Epigenetic factors influencing resistance to nuclear reprogramming

**DOI:** 10.1016/j.tig.2011.08.002

**Published:** 2011-12

**Authors:** Vincent Pasque, Jerome Jullien, Kei Miyamoto, Richard P. Halley-Stott, J.B. Gurdon

**Affiliations:** 1 Wellcome Trust/Cancer Research UK Gurdon Institute, The Henry Wellcome Building of Cancer and Developmental Biology, University of Cambridge, Tennis Court Road, Cambridge, CB2 1QN, UK; 2 Department of Zoology, University of Cambridge, Downing Street, Cambridge, CB2 3EJ, UK; 3 These authors contributed equally to this work

## Abstract

Patient-specific somatic cell reprogramming is likely to have a large impact on medicine by providing a source of cells for disease modelling and regenerative medicine. Several strategies can be used to reprogram cells, yet they are generally characterised by a low reprogramming efficiency, reflecting the remarkable stability of the differentiated state. Transcription factors, chromatin modifications, and noncoding RNAs can increase the efficiency of reprogramming. However, the success of nuclear reprogramming is limited by epigenetic mechanisms that stabilise the state of gene expression in somatic cells and thereby resist efficient reprogramming. We review here the factors that influence reprogramming efficiency, especially those that restrict the natural reprogramming mechanisms of eggs and oocytes. We see this as a step towards understanding the mechanisms by which nuclear reprogramming takes place.

## Routes towards nuclear reprogramming

The differentiated state of somatic cells in an organism is remarkably stable. Cells do not normally change from one differentiation pathway to another. However, adult somatic cells can be experimentally reprogrammed into other cell types, including pluripotent stem cells. By this route, the new cells obtained are genetically equivalent to the cells of origin and, similar to embryonic stem (ES) cells, can be induced to differentiate into any specialised cell type. Nuclear reprogramming (see Glossary) has great potential in terms of its medical application and, for this reason, many efforts have been made to increase its efficiency and to understand the mechanisms by which it occurs. Reprogrammed cells from patients can be used to study diseases in ways not previously possible and to design novel drug screens. Furthermore, reprogrammed cells could also provide a source of patient-matched replacement cells.

**Figure 1:**
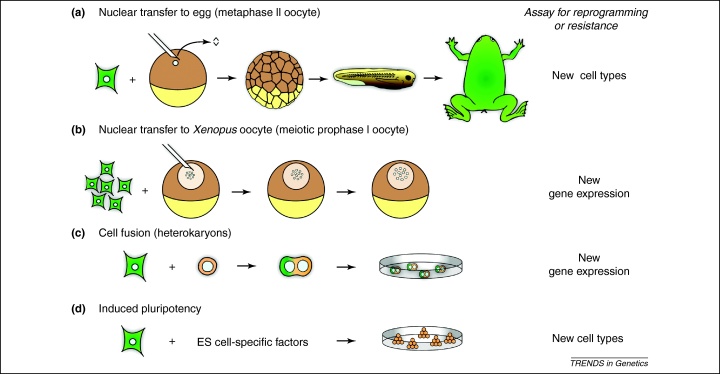
Different strategies induce nuclear reprogramming towards pluripotency. **(a)** During reprogramming by nuclear transfer to eggs, the nucleus of a cell is transplanted into an unfertilised egg whose own nucleus has been removed [1]. The resulting embryos, larvae and adults have the same genetic constitution as the donor nucleus. The animal and vegetal poles of the egg are shown in brown and yellow, respectively. **(b)** For nuclear reprogramming by nuclear transfer to *Xenopus* oocytes, multiple mammalian nuclei are transplanted into the nucleus (germinal vesicle) of a meiotic prophase I oocyte [5]. Transcriptional reactivation of previously silenced genes is induced without cell division or DNA synthesis, and no new cell types are formed. The animal and vegetal poles of the oocyte are shown in brown and yellow, respectively. **(c)** The nuclei of distinct cell types can be induced to reside within a common cytoplasm [8]. The fused cells form heterokaryons, in which the nuclei remain as separate entities, and these can be maintained by inhibiting cell division. **(d)** Pluripotency can be induced in cultured somatic cells by overexpression of embryonic stem (ES) cell-specific transcription factors or by overexpression of small noncoding RNAs together with histone deacetylases inhibitors [11,58]. The cells obtained are very similar to ES cells. Adapted, with permission, from [14].

Different systems have been used to reprogram cells (Figure 1). These include nuclear transfer to eggs and oocytes, cell fusion and overexpression of transcription factors. The nucleus of a specialised cell can be reprogrammed by somatic cell nuclear transfer (SCNT) to an enucleated egg (also called metaphase II oocyte; [1–3] but see also [4]). In this case, a somatic cell nucleus is reprogrammed by the egg to behave like the nucleus of an embryonic cell, and cells of the resulting embryo are pluripotent and able to differentiate into many, and sometimes all, cell types unrelated to the original donor nucleus (Figure 1a). The transcriptional state of somatic cell nuclei can also be reprogrammed by nuclear transfer to *Xenopus* meiotic prophase I oocytes (Figure 1b) [5]. Another route is to fuse two cells from different origins in such a way that the two nuclei of different cell types occupy the same cytoplasm; such fused cells form heterokaryons and cell hybrids (Figure 1c) [6–10]. In heterokaryons, the nuclei remain as separate entities within a common cytoplasm for a few days [8]. In proliferating cell hybrids, progression through the cell cycle causes the nuclei to fuse and give rise to synkaryons, which we do not discuss here. In heterokaryons, the nucleus of one donor cell is induced to express genes characteristic of the other donor cell, thereby providing an opportunity to investigate the mechanism of reprogramming. The cells fused can be of different species or differentiation state. For example, mouse ES cells can be fused to human fibroblasts [9]. Pluripotency can be induced in somatic cells by overexpression of a few transcription factors, originally *Oct4*, *Sox2* (both of which are required for pluripotency), *Klf4* and *c-Myc* (Figure 1d) [11]. The induced pluripotent stem (iPS) cells obtained have been well reviewed by others [12,13]. However, regardless of the system used, the proportion of nuclei or cells that are reprogrammed to new cell types is always low. This shows the resistance of somatic cells to reprogramming and reflects the stability of the differentiated state. Here, we concentrate on the epigenetic factors that promote or restrict the success or efficiency of nuclear reprogramming.

## Efficiency of nuclear reprogramming

**Figure 2:**
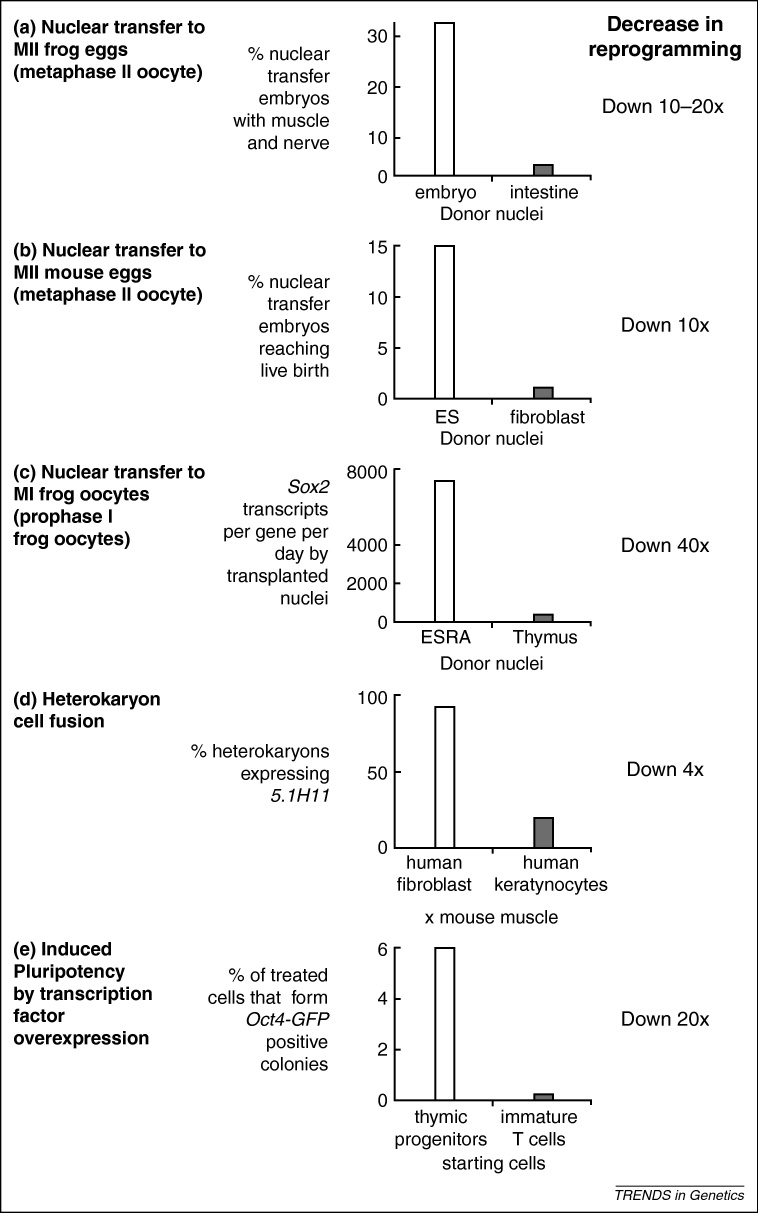
Resistance to reprogramming increases as cells differentiate. The extent of resistance to reprogramming (equivalent to a decrease in reprogramming efficiency) as cells differentiate, when tested by nuclear transfer **(a–c)**, cell fusion (heterokaryon) **(d)** and induced pluripotency **(e)**. Reproduced, with permission, from [15]**(a)**, [16]**(b)**, [5]**(c)**, [19]**(d)** (but also see [100,101]) and [22]**(e)**. Abbreviations: ES, embryonic stem; ESRA, retinoic-acid differentiated embryonic stem cells.

To understand the mechanisms of nuclear reprogramming and resistance to it, one needs to be able to judge the efficiencies of the various procedures; that is, the proportion of the starting cell population that responds to a reprogramming condition. If this proportion is very small, and if those cells that respond cannot be distinguished from those that do not, it is very hard to identify reprogramming factors and mechanisms. This is because most cells may not undergo reprogramming. There are striking differences in the speed and efficiency of reprogramming by different procedures and in resistance to it. There are two kinds of evidence for resistance to reprogramming. One comes from comparing nuclei from more or less differentiated cells; the other from comparing nuclei of different cell types. The efficiency of, and resistance to, nuclear reprogramming can be measured by many criteria. We have previously reviewed the criteria that can be used to judge reprogramming efficiency elsewhere [14]. Here, we only use the formation of different cell types or transcription of pluripotency genes as criteria (Figure 2).

When somatic cell nuclei are transplanted to enucleated eggs (in second meiotic metaphase), the efficiency with which new cell types are generated decreases by over 10-fold, as the donor cells from which nuclei are taken become more differentiated (Figure 2a). For example, the proportion of total nuclear transfers to *Xenopus* eggs that reach the swimming larval stage (with functional muscle and nerve) goes down from 35% with donor cells at the gastrula stage to 1.7% from tadpole intestinal epithelial cells, a decrease of up to 20 times [15]. In mice, the success of nuclear transfers from ES cells compared to those from adult fibroblasts decreases by 10-fold from 10–20% to approximately 1–2%, scored as the percent of total nuclear transfers that reach birth, as reviewed in [16] (Figure 2b), (but also see [17]). A similar decrease in success rate is seen with nuclear transfers to *Xenopus* oocytes (first meiotic prophase), when judged by pluripotency gene activation from transplanted nuclei. For example, the absolute number of *Sox2* pluripotency gene transcripts synthesised per transplanted nucleus per day goes from 7200 for differentiated ES cells to 160 for thymus, a decrease of 40-fold (Figure 2c) [5]. A similar decrease is seen for *Oct4* transcripts.

To determine the efficiency of reprogramming in cell fusion experiments, the most informative are those that result in heterokaryons. Efficiency can be assessed as the proportion of selected heterokaryons (1–2% of total fusions attempted) that express pluripotency genes, such as *Oct4*. Transcription of such genes can be detected in 70% of the heterokaryons (mouse ES and human fibroblasts) within one day [9], although the level of this expression is likely to be low (i.e. approximately 1% of the expression of these genes in ES cells) [18]. When one donor cell is highly differentiated, a lower proportion of heterokaryons activate some of the genes that are not expressed in the starting somatic cells [19]. For example, the proportion of heterokaryons that are induced to express the human muscle gene 5.1H11 6 days following fusion with mouse muscle cells is 95% for human lung fibroblasts, 60% for human keratinocytes and 25% for human hepatocytes (Figure 2d) [19]. We conclude that, in heterokaryons, as in nuclear transfers, nuclei from the most specialised cells are much more resistant to reprogramming than those of less specialised cells.

The overall efficiency of derivation of iPS cells by transcription factor overexpression is low (0.01% to approximately 6% of the treated cells) [11,12,20,21], but can be increased by various means, including noncoding RNAs, culture conditions, and so on. Transcription factor overexpression induces iPS cells approximately 20 times less often when immature T cells are compared to thymic progenitor cells, and approximately 300 times less efficiently when mature peripheral T cells are compared to thymic progenitors (Figure 2e) [22].

Resistance to reprogramming is also very evident when donor nuclei from different cell types are compared. In nuclear transfer to *Xenopus* oocyte experiments, ten times more transcripts of *Sox2* are made by transplanted nuclei of mouse embryonic fibroblasts (MEFs) than by those of the more differentiated C2C12 cells [5]. Conversely, the transcripts of *Oct4* and *Nanog* are five to eight times more abundant in transplanted C2C12 nuclei compared to nuclei of mouse embryo fibroblasts [5]. The difference between these two cell types in resistance is therefore at least 50-fold in respect of these genes. Because the reprogramming factors of an oocyte are the same for both kinds of nucleus, the 50-fold difference in responsiveness reflects the differential resistance of these genes in the two donor cell types.

Another aspect of resistance to reprogramming comes from the phenomenon of epigenetic memory, when different cell types are compared. In both nuclear transfer to egg experiments [23] and induced pluripotency work [24,25], reprogrammed nuclei and cells show persistent expression of genes that were active in donor cells, even though such genes are not normally transcribed in the derived cell types. In these cases, active genes resist a switch off after nuclear transfer or induced pluripotency, and this resistance can continue for numerous cell divisions.

The conclusion from this section is that there is a strong correlation between the more differentiated state of a cell and its resistance to reprogramming. Resistance is also seen when comparing the activation of quiescent genes in different cell types. We propose that this resistance to reprogramming reflects the stability of the differentiated state, and is the result of the progressive acquisition of epigenetic restrictions during embryonic development. We now review the epigenetic mechanisms that could account for this resistance and stability. Table 1 lists factors known to promote or restrict nuclear reprogramming.

## Epigenetic barriers to nuclear reprogramming

### Chromatin decondensation

The compaction of DNA in somatic cells is thought to be inhibitory to reprogramming. The first level of DNA compaction is defined by the wrapping of DNA around nucleosomes [26]. The presence of nucleosomes can prevent binding of certain transcriptional regulators, for example to DNA binding sites and, in particular, to large DNA recognition motifs. Therefore, efficient reprogramming requires mobilisation and remodelling of nucleosomes to allow transcriptional regulators to gain access to their genomic targets [27]. Consistent with this, most of the factors with the ability to promote access to gene regulatory regions have been found to be able to increase reprogramming efficiencies (Table 1) [28].

**Figure 3:**
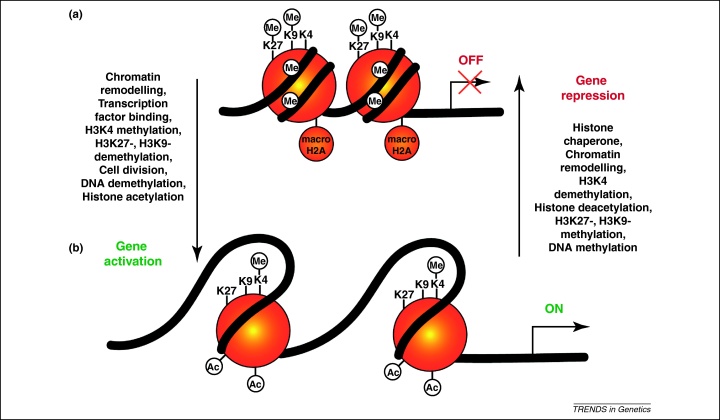
Hypothetical model of chromatin state changes at gene regulatory regions during reprogramming and differentiation. Epigenetic reprogramming of chromatin states requires several events, some of which are summarised here. A fully repressed gene **(a)** must be remodelled to evict repressive nucleosomes, which may contain histone variants such as macroH2A and multiple repressive histone modifications. Once accessible, regulatory regions may be bound by transcriptional regulators with the ability to recruit activities, such as H3K4 methyltransferases. Loss of repressive histone modifications, such as H3K9me2/3, H3K27me2/3 and DNA methylation and demethylation may occur actively or passively through cell divisions. Histone acetylation also strongly increases transcriptional activity **(b)**. The opposite route may lead to transcriptional silencing of differentiation genes during reprogramming towards pluripotency, or silencing of pluripotency genes during cell differentiation. The steps represented may occur simultaneously and/or in a different order according to the gene and system considered. The order of the epigenetic events that occur during nuclear reprogramming may not be in the exact reverse order of the events that occur during cell differentiation.

As cells differentiate, their chromatin becomes increasingly condensed. Nuclear volume is indicative of the average extent of chromatin condensation. We estimate the volume of a nucleus (inversely related to condensation) in lymphocytes, non-mammalian red blood cells, and sperm, to be three, eight or 100 times respectively, smaller than that of an ES cell. In all nuclear transfer experiments, both in eggs and oocytes, a nuclear volume increase of 10–30-fold accompanies new gene transcripts [29], chromosomal proteins leave the nucleus and chromosomal protein mobility is increased [30]. Likewise, in heterokaryon experiments, similar changes follow cell fusion [6,7,31]. However, changes in nuclear volumes are not sufficient for gene reactivation because Polycomb-deficient ES cells do not induce pluripotency gene reactivation when fused to human B-lymphocytes but nuclear volume changes remain unperturbed [32]. In Figure 3, we present a hypothetical model of chromosomal changes associated with nuclear reprogramming.

Two components of eggs and oocytes that seem particularly important for chromatin decondensation are nucleoplasmin (a chaperone of histones H2A and H2B) [33], and a special oocyte-specific linker histone named B4 for amphibians or H1foo for mammals [34,35]. B4 incorporation into nuclei transplanted to *Xenopus* oocytes is complete in few hours, and is necessary for pluripotency gene activation [30]. We interpret these results as indicating an opening of chromatin structure to expose those genes that are quiescent in somatic cells to the transcriptional-activating components of eggs and oocytes. In the case of eggs and oocytes, the opening up of chromosome structure after nuclear transfer may well be global; that is, not gene specific. Supporting this view is the fact that a wide range of genes, including lineage-specific genes normally expressed in muscle, nerve, and so on, start to be transcribed in somatic nuclei transplanted to *Xenopus* oocytes [36]. Although reprogramming to induced pluripotency may be mechanistically different, the chromatin remodelling enzyme Chd1 has been shown to be important for the induction and maintenance of pluripotency by promoting an open chromatin state [37]. Chromatin remodellers Brg1 and Baf155 have been found to increase the efficiency of *Oct4-GFP* reactivation during induction of pluripotency from mouse embryonic fibroblasts (MEFs) [38], in addition to egg extract work [39].

We suggest that chromatin decondensation and loss of chromosomal proteins is a primary event that is required, but not sufficient for reprogramming and therefore counteracts differentiation-related resistance. Different reprogramming systems seem to use different ways to promote chromatin decondensation.

### DNA demethylation

The best-known epigenetic mechanism that imposes a roadblock to nuclear reprogramming is DNA methylation. Reprogramming by nuclear transfer, by cell fusion and by induced pluripotency is associated with a global reversal of DNA methylation so that somatic nuclei closely resemble those of ES cells [9,24,40–42]. DNA demethylation of repressed genes is required for gene reactivation during reprogramming [9,43,44] and the failure of this has been correlated with poor development of cloned embryos [45]. Derivation of mouse ES cells by nuclear transfer is more efficient when the donor nuclei lack DNA methyltransferase 1 (Dnmt1), an enzyme needed for DNA methylation [46] and the transient inhibition of Dnmt1 has also been found to help the transition from partially to fully reprogrammed iPS cells (Table 1) [25,44]. Therefore, DNA demethylation is a key step during nuclear reprogramming, although it is not clear how much of it results from active DNA demethylation versus passive loss through cell divisions. Eggs and oocytes seem to induce DNA demethylation more efficiently than does transcription factor-based reprogramming [24]. The mechanisms of active DNA demethylation are currently being unravelled and include hydroxylation of methylated cytosines by Tet enzymes and/or deamination by AID/APOBECs followed by DNA repair [9,47,48].

The whole-genome profiling of DNA methylation in iPS cells and in ES cells derived by nuclear transfer reveals that an incomplete reversal of DNA methylation takes place in reprogrammed cells, indicating that, in such cells, reprogramming is not fully efficient [24,49,50]. Incomplete DNA demethylation clearly contributes resistance to reprogramming.

It is important to appreciate that there are instances in which a resistance to reprogramming is not fully explained by DNA methylation alone. The inactive X chromosome of female mammalian cells is commonly associated with methylated DNA. By contrast, the inactive X chromosome of female mouse epiblast stem cells is methylated yet it can be reactivated by nuclear transfer to *Xenopus* oocytes, whereas the inactive X of MEFs, also methylated, is resistant to reactivation [51]. DNA methylation only restricts transcription in specific chromatin contexts [52], for example in promoters, where it may directly prevent transcription factor binding or promote DNA compaction. Furthermore, methylated plasmid DNA is perfectly well transcribed in *Xenopus* oocytes until it becomes chromatinised and hypoacetylated through the recruitment of histone deacetylases (Hdac) [53]. The main conclusion here is that DNA demethylation takes place during nuclear reprogramming, but is incompletely effective and so can cause resistance to successful reprogramming.

### Histone modifications and histone variants

Histone tails are subject to numerous post-translational modifications that are important for the regulation of chromatin structure and gene expression [54]. Histone deacetylation commonly accompanies gene repression in differentiated cells. Inhibitors of Hdac, including valproic acid (VPA) and trichostatin A (TSA) often promote the success of nuclear reprogramming (Table 1) [55–57]. For example, the frequency of obtaining cloned offspring by nuclear transfer to mammalian eggs is improved up to fivefold by Hdac inhibition [55,56]. Gene reactivation is also enhanced by Hdac inhibition in induced pluripotency experiments [57]. The downregulation of Hdac2 allows the induction of pluripotency from MEFs solely by expression of *miR*302/367 [58]. It may be that an inhibition of differentiation programs, together with appropriate culture conditions, may be sufficient for the induction of pluripotency. In *Caenorhabditis elegans*, expression of the gustatory neurons inducing transcription factor CHE-1 together with either Hdac inhibition or the deletion of the histone chaperone lin-53 allows reprogramming of germ cells into neurons [59]. No other cell type is affected by CHE-1 overexpression, an indication that, in *C. elegans*, certain chromatin factors can provide a cell type-specific resistance to reprogramming [59]. Altogether, inhibiting Hdac activity generally improves reprogramming.

The ‘active’ histone mark H3K4me2/3 is important for transcription initiation and activity [60] and is associated with transcriptional gene reactivation after somatic cell nuclear transfer to *Xenopus* oocytes [61]. In agreement with this, in induced pluripotency experiments, H3K4me2 is deposited before the first cell division and prior to signs of transcriptional activation at a subset of genes [62]. It is thought that this event may increase accessibility of regulatory regions of DNA. The Trithorax protein Wdr5, an effector of H3K4 methylation, was shown to be required for the formation of iPS cells (Table 1) [63].

Other histone marks are associated with gene repression and undergo large changes during nuclear reprogramming. The maintenance of large chromatin blocks containing H3K9me2 (LOCKs) is associated with epigenetic memory, which increases resistance to nuclear reprogramming [64,65]. The H3K9me2/3 methyltransferase G9a has been shown to restrict reprogramming in part through DNA methylation [66]. In agreement, the expression of the H3K9me3 demethylase *Kdm3a* or G9a removal, both increase the efficiency of reprogramming following nuclear transfer and cell fusion (Table 1) [66,67]. H3K9me3 inhibitors, such as BIX-01294, also increase the efficiency of iPS cells derivation [68].

The histone variant macroH2A is commonly associated with heterochromatin in vertebrates and is usually incorporated after gene silencing has been induced [69]. Interestingly, eggs contain an activity that removes macroH2A from the nucleus after fertilisation and after nuclear transfer [70,71]. The knock-down of macroH2A in MEFs increases the transcriptional reprogramming efficiency of *Oct4* and *Sox2* in *Xenopus* oocytes [51]; therefore macroH2A seems to cooperate with other silencing mechanisms to maintain the repressed state of genes in somatic cells and so helps to account for resistance to reprogramming. It is thought that macroH2A may directly restrict reprogramming by preventing transcription factor binding [72], by preventing histone acetylation, and by recruiting Hdacs [73,74]. macroH2A also seems to reduce the affinity of SWI/SNF remodelling complexes for chromatin [75], these complexes being thought to be required for nucleosome mobility and hence for access of factors to repressed genes.

## Transcriptional components that promote or restrict reprogramming

In reprogramming experiments when new cell types are not formed (*Xenopus* oocytes and heterokaryons), the transcription of pluripotency and other genes is used as a measure of successful reprogramming (Figure 1). In nuclei transplanted to *Xenopus* oocytes, the rate of transcription of such genes increases greatly from an undetectable level in donor cells to 1200 (or 170) new transcripts per gene per day for *Sox2* (or *Oct4*) [5]. The mechanism of this transcriptional activation is known to be related to an exceptionally high content of transcriptional components in *Xenopus* oocytes. This includes enough polymerase II for the transcription of over 10 000 somatic nuclei [76,77], as happens when normal *Xenopus* embryos reach the stage of transcriptional activation (the blastula stage) [78]. All polymerase II in the blastula is thought to be derived from the oocyte content [77]. Histone H3.3 is closely associated with active transcription [79] and is exceptionally abundant in oocytes (G. Almouzni, personal communication). Also, a high content of polymerised actin is characteristic of the oocyte germinal vesicle; it is present in somatic nuclei that are reprogrammed by *Xenopus* oocytes, and is required for successful transcriptional reprogramming [80]. Therefore the exceptionally high content of transcriptional components in the oocyte germinal vesicle helps to account for the transcriptional activation of genes in transplanted nuclei.

We think that the resistance of somatic nuclei to transcriptional reprogramming by oocytes can be explained by the condensed state of chromatin. It is known that the rate of transcription increases enormously as the chromatin of nuclei transplanted to *Xenopus* oocytes becomes decondensed, and does so in direct proportion to nuclear volume increase [81]. As the chromatin of nuclei becomes decondensed in injected oocytes, polymerase II and other transcriptional components gain access to previously quiescent genes.

The high content of histone H3.3, a transcription-related histone variant, may account for the phenomenon of epigenetic memory, mentioned above, in which somatic nuclei transplanted to *Xenopus* eggs resist the switching off of genes active in donor cells [23]. For example, muscle-specific genes are actively transcribed in the nuclei of muscle cells. The unusually high H3.3 content in eggs may promote the continuing transcription of such genes in developing embryos in non-muscle cells, in a way that would not happen in sperm after fertilisation, because sperm nuclei do not have active muscle genes.

There is recent evidence that numerous noncoding RNAs are important regulators of transcriptional and epigenetic states [82]. The noncoding RNA *Xist* plays a role in inducing the transcriptional inactivation of a female mammalian X chromosome [83]. In the mouse, half of the genes that resist reprogramming in nuclear transfer embryos are located on the inactive X chromosome [64,84]. These embryos aberrantly express *Xist* on the active X chromosome, leading to aberrant inactivation of X-linked genes [64]. In this case, resistance to gene activation is caused by the mis-regulation of a noncoding RNA that now guides the silencing machinery to chromatin. The deletion of one copy of *Xist* from donor nuclei is sufficient to decrease resistance and so increase the efficiency of cloned offspring derivation by nuclear transfer. It seems probable that other noncoding RNAs, short or long, may also contribute resistance to reprogramming (Table 1). One study identified a set of long noncoding RNAs upregulated during reprogramming to pluripotency; one of these facilitates reprogramming [85]. Several groups have reported that interference with the RNAi machinery can significantly alter reprogramming, and that the introduction of specific miRNAs can help iPS cell derivation [58,86–88].

## Cell division helps but is not required

When new cell types are formed after reprogramming in nuclear transfer to eggs and in induced pluripotency experiments, extensive cell division always takes place before new cell types appear. It has been speculated that cell division might contribute to reprogramming, possibly through the replacement of chromosomal proteins at mitosis or by the assimilation of new chromosomal proteins during DNA synthesis [89]. However, reprogramming as judged by new gene transcription clearly does not require cell division or DNA synthesis, because these do not take place in oocyte nuclear transfer or in heterokaryon experiments [5,19,90]. It is also known that DNA demethylation can occur in the absence of cell division [9,43,91]. In another example, the conversion of *C. elegans* Y epithelial cells into motoneurons can occur in the absence of cell division [92]. Nevertheless, cell divisions seem to facilitate reprogramming in systems where they occur and may be required for a full level of transcription and for the generation of new cell types [93,94]. The resetting of replication origins from a somatic type to an embryonic one is seen when somatic nuclei are incubated in oocyte extract, suggesting that this is important for reprogramming by nuclear transfer [95,96].

## Concluding remarks and future perspectives

The cytoplasm of eggs, somatic and pluripotent cells, or ectopically expressed factors, can reprogram the nucleus of many kinds of somatic cell, so that gene expression (of these nuclei) is switched to that characteristic of the initial cytoplasmic cell type. Mechanisms of reprogramming include chromatin decondensation and remodelling, DNA demethylation, histone modifications and changes in the rate of transcription of many genes (including those required for pluripotency). As cells become more differentiated, their nuclei become increasingly resistant to reprogramming. Resistance seems to depend on the acquisition of a combination of several epigenetic factors, each of which contributes to the stability of the differentiated state. Eggs, oocytes, somatic cells or ES-cell-specific factors are incompletely efficient at reversing these stabilising factors.

We think that reprogramming may be different for induced pluripotency by transcription factor overexpression compared to nuclear transfer and cell fusion. The former may be achieved by a stochastic vacancy of transcription factor binding sites in otherwise undisturbed chromatin [94,97–99]. Nuclear transfer and cell fusion do not involve transcription factor overexpression, but need chromatin remodelling.

Although different reprogramming systems may use different routes to achieve reprogramming, we think that five steps are required for the complete switch from a differentiated somatic cell to an embryonic cell or to an unrelated differentiated cell by nuclear transfer, cell fusion or induced pluripotency (Box 1). In the case of nuclear transfer to second meiotic metaphase eggs and induced pluripotency by transcription factor overexpression, all five steps take place in an overlapping time sequence. By contrast, these reprogramming steps seem to be separate in nuclear transfer to *Xenopus* oocyte (first meiotic prophase) experiments in which only steps i–iii take place. Cell division (step iv) and suppression of competing pathways (step v) occur only as eggs divide and as different cell lineages begin to appear. However, resistance to reprogramming is clearly evident in oocyte nuclear transfer experiments in the absence of cell division. We conclude that resistance to reprogramming in nuclear transfer experiments is caused, at least in part, by incomplete chromatin decondensation, incomplete removal of differentiation chromatin marks and, hence, by incomplete transcriptional activation. As cells differentiate, they progressively acquire more and more epigenetic marks that restrict reprogramming. Although oocytes are endowed with components that promote nuclear reprogramming, it may be that the process of cell differentiation progressively compacts the chromatin of specialised cells, in particular that of quiescent genes, so that access to important genes is a slow process.

A mechanistic understanding of the epigenetic factors that restrict reprogramming in different systems is only starting to emerge. Identifying the epigenetic factors and understanding the mechanisms that restrict somatic cell nuclear reprogramming is one important aim for the reprogramming field, in addition to finding ways of removing these restrictions efficiently from somatic cells. This will be required to generate efficiently useful replacement (stem) cells to be used for therapy.

## Figures and Tables

**Table 1:** Cellular factors that influence nuclear reprogramming

	Factors	System	Refs
**Promote**			
Transcription factors	Oct4, Sox2, Klf4, c-Myc, Nanog	Induced pluripotency	[11,12,102]
Chromatin decondensation and remodelling	Histone B4, nucleoplasmin	Nuclear transfer	[30,33]
	Brg1, BAF155, Chd1	Induced pluripotency	[37–39]
DNA demethylation	AID	Cell fusion	[9]
	Tet3	Nuclear transfer	[47]
H3K9me2/3 demethylation	Kdm3a, Kdm4c	Cell fusion	[67]
Trithorax proteins	Wdr5	Induced pluripotency	[63]
Polycomb proteins	PRC2: Eed^a^, Ring1b^a^	Cell fusion	[32,103]^a^
Cell division	Mitosis	Induced pluripotency	[89,93,94]
	DNA replication	Nuclear transfer	[89,95,96]
Small noncoding RNAs	miR-291-3p, miR-294, miR-295, miR-93, miR-106b, miR302/367	Induced pluripotency	[58,86–88]
Long noncoding RNAs	Long noncoding RNA-RoR	Induced pluripotency	[85]
**Restrict**			
DNA methylation	Dnmt1	Nuclear transfer	[43,46]
	Dnmt1	Induced pluripotency	[25,44]
Histone deacetylation	Hdac	Nuclear transfer	[51,55,56]
	Hdac	Induced pluripotency	[57,58]
H3K9me2/3 methylation	LOCKs, G9a	Nuclear transfer	[64,66]
	G9a	Induced pluripotency	[68]
	G9a	Cell fusion	[67]
Histone variants	macroH2A	Nuclear transfer	[51]
			
			

a Eed and Ring1b were demonstrated to be required in ES cells for their ability to induce transcriptional reprogramming of pluripotency genes following fusion with human lymphocytes [32].

## Box 1 Nuclear reprogramming events required to yield new cell types

(i) Chromatin decondensation
(ii) Loss of differentiation marks
(iii) Transcriptional activation
(iv) Cell division
(v) Suppression of competing cell lineages*

In the case of nuclear transfer to second meiotic metaphase eggs and induced pluripotency by transcription factor overexpression, all five steps take place in an overlapping time sequence. By contrast, these reprogramming steps seem to be separate in nuclear transfer to *Xenopus* oocyte (first meiotic prophase) experiments in which only steps i-iii take place. The five steps shown may occur in a different order.

## Glossary

DNA methylation: addition of a methyl group to a cytosine base residue in DNA, often localised next to a guanine base. Methylated cytosines can be further modified by hydroxylation. Methylated cytosines can lead to the recruitment of specific methyl DNA-binding proteins, which may lead to transcriptional repression.
Epigenetic: heritable changes in gene expression that do not involve changes in the DNA sequence.
Epigenome: the epigenetic state of the genome.
Histone modifications: histones are the basic unit of the nucleosome and are subjected to a large number of post-translational modifications, which play an important role in regulating chromatin structure and, hence, regulation of gene expression. Both histone tails and core residues can be subjected to modifications as diverse as acetylation, methylation, phosphorylation and ubiquitinylation, to cite a few.
Histone variants: most histone variants are distinguishable from core histones by a few amino acid changes or by a larger non-histone domain. These divergences confer important functions on histone variants and therefore add to the complexity of epigenetic regulation. Histone variants can replace core histones in a nucleosome.
Induction of pluripotency: refers to pluripotent stem cells that have been reprogrammed from somatic cells by forced expression of specific transcription factors.
Noncoding RNAs: RNAs that are encoded by genes, but are not translated into proteins. Instead, their structure allows them to interact functionally with various biochemical processes, such as translation, transcription and chromatin structure.
Nuclear reprogramming: changes in gene activity that are induced experimentally by exposing a nucleus to a new environment.
Nuclear transfer: the transfer of one or multiple cell nuclei into eggs or oocytes. The transplantation of a somatic cell nucleus into an enucleated egg (metaphase II oocytes) can lead to the development of a cloned embryo. The technique is often referred to as SCNT. The transfer of multiple nuclei into the nucleus of a *Xenopus* oocyte (meiotic prophase I) leads to transcriptional reactivation of quiescent genes.
Pluripotency: the capacity of a cell to generate most of the cell lineages of the body, including germ cells but excluding extra-embryonic lineages.
Somatic memory: persistent characteristic of differentiated cells present in reprogrammed cells. The memory results from the incomplete erasure of the somatic cell epigenome.
Transcription factors: proteins that bind to specific DNA sequences to control gene expression. Transcription factors can form multiprotein complexes and bind regulatory regions to control the recruitment and activity of RNA polymerases.

## Footnotes

* For cells to follow a differentiation pathway correctly, other competing pathways may need to be suppressed.
